# A CARE-compliant article: A case report of retroperitoneal endometrial stromal sarcoma with multiple pulmonary metastases and literature review

**DOI:** 10.1097/MD.0000000000039093

**Published:** 2024-08-09

**Authors:** Yaoyao Fang, Xiaoli Zhou, Jing Bai, Qiuju Lin, Yingchun Zheng, Sijia Bo, Lei Sui, Ting Zhu, Nuo Bai, Li Sun

**Affiliations:** a School of Clinical Medicine, Shandong Second Medical University, Weifang, China; b Department of Internal Medicine, Lixia District People’s Hospital, Jinan, People’s Republic of China; c Department of Maternity, Jinan Maternity and Child Care Hospital Affiliated to Shandong First Medical University, Jinan Maternity and Child Care Hospital, Jinan, People’s Republic of China; d First Department of Gynecology Oncology, Qingdao Central Hospital, University of Health and Rehabilitation Sciences (Qingdao Central Hospital), Qingdao, China.

**Keywords:** adenomyosis, endometrial stromal sarcoma, pulmonary metastases, retroperitoneum, subtotal hysterectomy

## Abstract

**Rationale:**

Endometrial stromal sarcoma is an extremely rare mesenchymal neoplasm occurring in the extrauterine. Retroperitoneal endometrial stromal sarcoma with multiple pulmonary metastases, in particular, is extremely rare.

**Patient concerns:**

Forty-seven-year-old woman (gravida 3, para 2), was referred to our institution with complaints of fever.

**Diagnoses:**

Ultrasound and computed tomographic imaging of the abdomen identified the presence of masses in the pelvic region. Additionally, computed tomographic scans and X-ray evaluations of the thorax detected dispersed masses in both the lungs and pelvic area. Histopathological analysis of the pelvic region indicated the presence of low-grade endometrial stromal sarcoma. A computed tomography-guided pneumocentesis was conducted to further characterize the pulmonary lesions, confirming the diagnosis of low-grade endometrial stromal sarcoma.

**Interventions:**

The patient underwent tumor resection, subsequent treatment with Medroxyprogesterone acetate for 6 months, received microwave ablation for multiple lung metastases, PARP1 inhibitor for 4 courses, and has been undergoing chemotherapy (epirubicin/ifosfamide) up to the present time.

**Outcomes:**

Partial remission was obtained after the above treatment and this patient is now still alive maintaining for 3 years.

**Lessons:**

The identification and management of this disease remain a significant challenge due to its low prevalence. Further research involving a larger number of cases is necessary to ensure consistency in diagnosis and to establish effective treatment guidelines.

## 1. Introduction

Endometrial stromal sarcoma (ESS) is a rare uterine tumor that accounts for 0.2% to 1% of all uterine malignancies and 15% of uterine sarcoma.^[1]^ Based on the mitotic activity, ESS is classified as low-level and high-grade. However, according to the 2003 World Health Organization classification, it is considered to be undifferentiated uterine sarcoma as it exhibits destructive myometrial invasion rather than involvement of lymph node.^[2]^ Low-grade ESS (LG-ESS) is characterized as slow growth and a tendency to relapse lately, with a 5-year survival rate of 80% to 100% and a recurrence rate of approximately 50%. Extrauterine ESS is extremely rare and the most common regions are ovaries, peritoneum, rectovaginal septum, vagina, and colorectal serosa.^[3,4]^ Here, we report a case that was diagnosed as retroperitoneum LG-ESS primarily arising from endometriosis lesion with pulmonary metastases. This patient received surgery and subsequent adjuvant chemotherapy, hormonotherapy, microwave ablation, and targeted therapy.

Through reviewing the previous reported cases, this is the 6th case of the ESS which is primarily located in retroperitoneum, and the first case arising from adenomyosis with pulmonary metastases.

## 2. Case presentation

**Figure 1.**
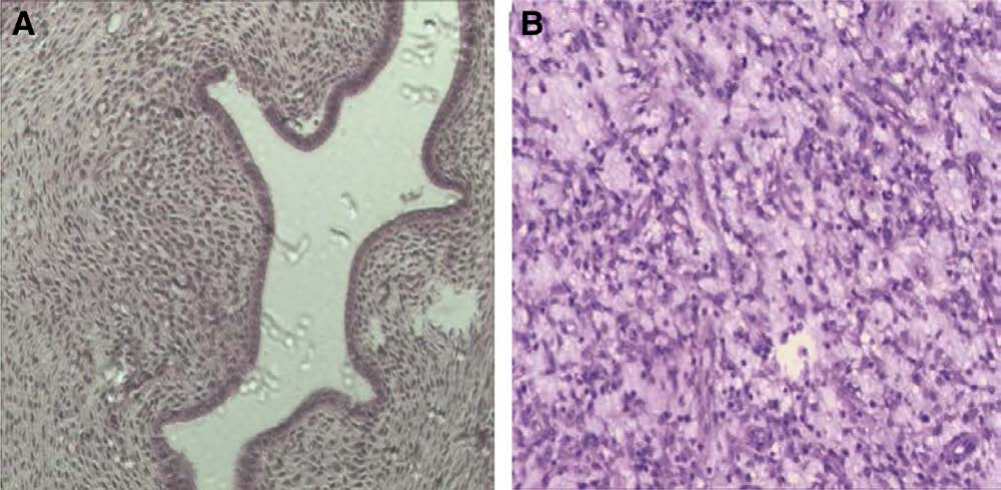
The pathological examination of subtotal hysterectomy shows adenomyosis (A). Pathologic examination for specimens from pelvic shows low-grade ESS (B). (A) Low power view, HE × 100. (B) Higher power view. The sarcoma component infiltrated into adjacent myometrium HE × 400. The tumor cells showed pronounced cytologic atypia with undifferentiated bizarre or giant sarcoma cells. Higher power view HE × 400.

**Figure 2.**
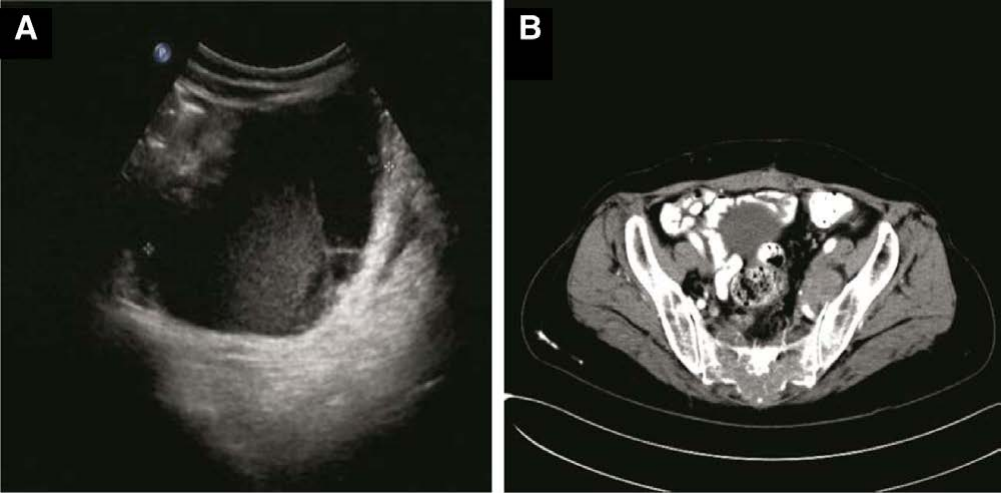
Abdominal ultrasound (A) and computed tomographic scan (B) revealed pelvic masses with a diameter of 8 cm × 6 cm × 4 cm tumor in left pelvic and another one of 6 cm × 4 cm × 3 cm closely relative to posterior wall.

**Figure 3.**
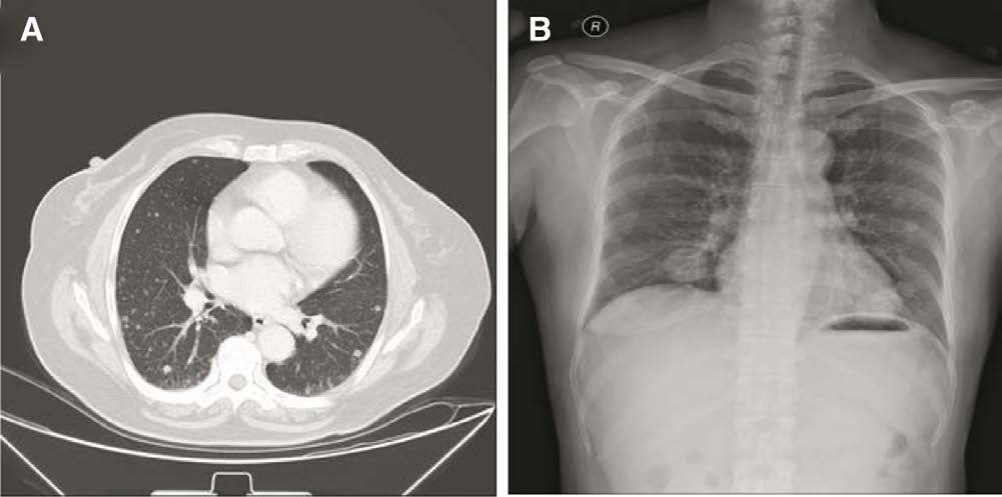
Computed tomographic scan (A) and X-ray examination (B) of the thorax revealed scattered masses in lung.

**Figure 4.**
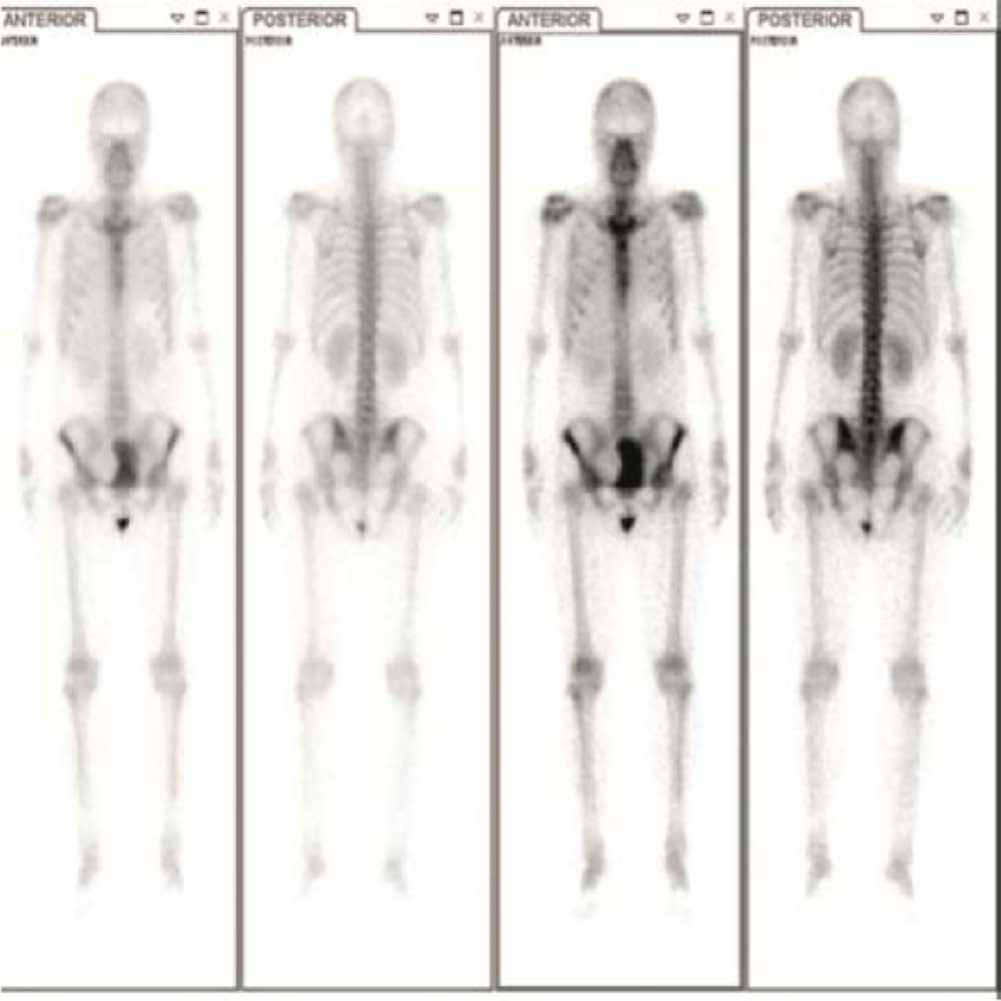
A radionuclide bone scanning showed no radioactive concentration.

Forty-seven-year-old woman (gravida 3, para 2), presented with fever in August 2016. She had a history of adenomyosis and had received laparoscopic subtotal hysterectomy for 4 years (Fig. 1A). Ultrasound and computed tomographic scan of abdomen revealed pelvic masses (Fig. 2). Computed tomographic scan and X-ray examination of thorax found scattered masses in the lung and pelvic (Fig. 3). The levels of serum carcinoembryonic antigen and CA125 were within normal values. A radionuclide bone scanning showed no radioactive concentration (Fig. 4). The patient underwent laparotomy with pelvic mass resection, bilateral salpingo-oophorectomy, pelvic lymph node dissection, omentectomy, and appendicectomy. During the process of surgery, there appeared to be pelvic masses (left diameter: 6 cm and right diameter: 8 cm) in retroperitoneal of pelvic. Further analysis of these granulations found mild cytologic atypia and myxoid matrix. In addition, abdominal dropsy cytology revealed no lesion. Histopathologic examination of pelvic showed low-grade ESS (fibromyxoid subtype) (Fig. 1B). A computed tomography-guided pneumocentesis was performed to define the pulmonary lesions and found to be LG-ESS.

The patient received 6 courses of platinum-containing combination chemotherapy (paclitaxel/carboplatin) and subsequent treatment with medroxyprogesterone acetate for 6 months, and obtained a partial remission.

**Figure 5.**
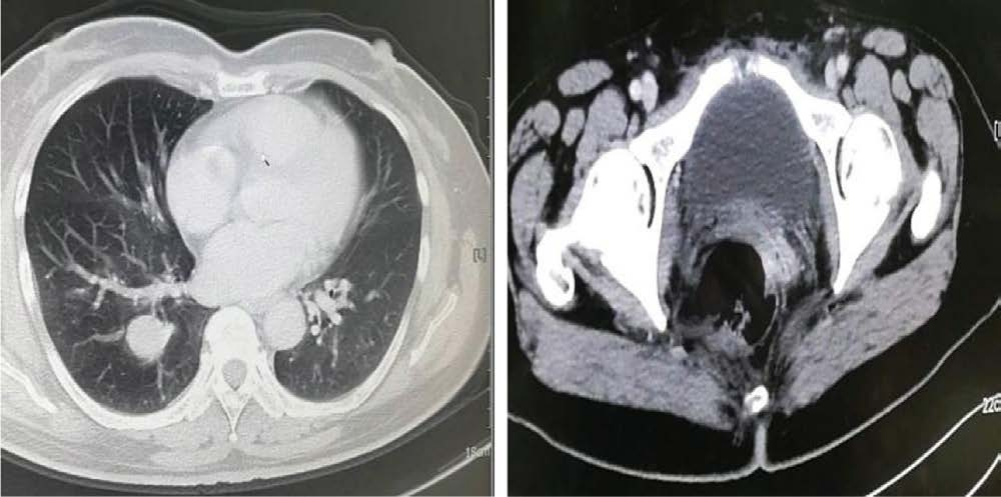
Computed tomographic scan examination revealed relapse in lung and pelvis.

The patient stopped taking medroxyprogesterone acetate privately due to increased weight. She received computed tomographic scan of thorax, abdomen, and pelvic in December 2017 and found abdominal aortic and pelvic lymph node enlargement and pulmonary metastases progressed (Fig. 5). Then, the patient received microwave ablation for multiple lung metastases.

The patient participated in a clinical trial for SOMCL-9112 (PARP1 inhibitor) for 4 courses in August 2018 at Shanghai Cancer Hospital, and stopped taking this medication due to tumor propagation and severe myelosuppression. Then she returned to our center and received 2 courses of chemotherapy (epirubicin/ifosfamide). After chemotherapy, this patient achieved partial remission. Now, the patient is still receiving chemotherapy.

The specimen of subtotal hysterectomy was analyzed by our center and Beijing Medical Third Hospital, and revealed adenomyosis with slightly active interstitial hyperplasia. However, no LG-ESS tumor cell was found.

The specimen consisted of 2 tumors in the retroperitoneal of pelvic, bilateral salpingo-oophoron, appendix, and omentum. A diameter 8 cm × 6 cm × 4 cm tumor in left pelvic and another one with a size of 6 cm × 4 cm × 3 cm underwent surgical excision. Tumor invasion was not observed in the bilateral salpingo-oophoron, appendix, and omentum.

Microscopic analysis revealed a typical tongue-like growth of tumor nodules. These nodules were composed of densely packed, plump spindle cells in short fascicles, interspersed with prominent small arterioles. The tumor cells resembled those of normal endometrial stroma in proliferative phase, with scanty ill-defined cytoplasm and round or ovoid shape of nuclei with dispersed chromatin. The cells exhibited little nuclear pleomorphism and few mitotic figures were found with the mitotic count being <1 in 10 high-power fields. There was 1 area of epithelial-like configuration. The histologic features were fitted into the typical low-grade ESS.

There was no lymph node metastasis, and all surgical margins were free of disease. Tumor cells were strongly positive for CD10, estrogen receptor, and progesterone receptor, whereas, a few tumor cells in an area of epithelial-like configuration were positive for Desmin. The follow-up time was 3 years and no signs of tumor recurrence were found.

## 3. Discussion

Malignant transformation of endometriosis has been well documented since it was first reported by Sampson,^[5]^ who recommended 3 criteria for a definitive diagnosis of malignancy arising from endometriosis: close proximity of benign endometriosis to the malignant tumor, no other primary sites identified, and tumor histology compatible with an endometrial primary. Scott^[6]^ suggested that a more stringent qualification should be applied, requiring that microscopic benign endometriosis was contiguous with malignant tissues. For the extra ovarian case, adequate evidence for such an association is the coexistence of tumors and endometriotic tissues, even without observable continuity, if the 2 processes appear in an uncommon site or at an unusual age, and the malignant tumor is of a histologic type that has been well established to arise from endometrial-type tissue.^[7]^ These less strict criteria are justified because transitional areas between endometriosis and cancer can be destroyed by the growth of tumors and therefore these were found in only 5% to 10% of cases.^[5]^ Our case is consistent with these criteria.

The most common histologic type of cancer arising from endometriosis is endometrioid adenocarcinoma, and ESS is rare. ESS is extremely uncommon in extrauterine sites of malignant transformation of endometriosis.^[8]^ In the review of preneoplastic and neoplastic changes in extragonadal endometriosis, Kim et al^[9]^ found about 80% of endometriosis-associated malignancies are localized in the ovary, whereas extragonadal sites are affected in 20% to 25% of all cases.

We reviewed similar cases through searching published reported in PubMed, and found only 5 cases (Table 1).^[3,4,10–12]^ The age of patients ranged from 43 to 76. The most common clinical symptoms are abdominal discomfort and pelvic mass without metastasis. Fever as the first presenting symptom of retroperitoneal ESS has never been reported, probably due to tumor necrosis or infection. The association between retroperitoneal ESS and endometriosis is reported for the first time to our knowledge.

**Table 1:** Clinicopathological features of 5 patients with retroperitoneal ESS.

No	Age	Clinical presentation	Endometriosis	Treatment	Metastasis	Recurrence	Follow-up (mo)
1	76	Pelvic mass	–	ST	–	–	ANED (36)
2	57	Pelvic mass	–	Unresect	–	–	DOD (UNK)
3	71	Abdominal fullness	–	ST	–	–	ANED (91)
4	43	Menorrhagia, pelvic mass	–	ST	–	–	ANED (15)
5	60	Abdominal pain	–	ST	UNK	UNK	UNK

ANED = no evidence of disease, DOD = dead of disease, ESS = endometrial stromal sarcoma, ST = surgical treatment, UNK = unknown, Unresect = unresectable.

ESS is debated diagnosis of mesenchymal neoplasms and is challenging for preoperative diagnosis. Most of the mesenchymal neoplasms (fibromatosis, schwannoma, and leiomyoma) can be immediately excluded from the differential diagnosis based on the histologic features. However, given their variable gross and histologic appearances, sex cord-stromal tumors can be misdiagnosed with ESS. In contrast to ESS, these tumors tend to be well-circumscribed with broad, pushing borders and rare vascular invasion. The cells are arranged in short fascicles with a vaguely organoid arrangement reminiscent of smooth muscle neoplasms. Nuclear atypia and pleomorphism may be marked. ESSs have characteristically invasive tongues of tumors at the periphery and are usually composed of short regular fascicles or sheets of monomorphic plump spindle cells. The presence of prominent arterioles and extensive vascular invasion should rule out the diagnosis of stromal tumor. Finally, immunohistochemical staining is useful in distinguishing between these entities, as stromal tumors are well known to diffuse staining for CD117 and CD34^[13]^ and ESS for estrogen and progesterone receptors. CD10 is a well-known positive diagnostic marker for ESS and our case was strongly positive for CD10, estrogen receptor, and progesterone receptor.

The management of extrauterine ESS is difficult, but primary surgical treatment with complete resection should be performed when feasible.^[14]^ The application of adjuvant treatment, including chemotherapy, radiation therapy, and endocrine therapy remains controversial.^[15]^ One of all 5 cases has not been resectable and died, and the others all receive surgical treatment without adjuvant therapy. Follow-up time ranges from 15 to 91 months and all patients who received surgery have no evidence of disease. As presentation of pulmonary metastases, our patient received adjuvant therapies after surgery, and partial remission was obtained after chemotherapy indicating that paclitaxel/carboplatin and epirubicin/ifosfamide might be effective regimens for ESS. This patient is now still alive maintaining for 3 years.

## 4. Conclusions

In summary, retroperitoneal ESS is rare, and its association with endometriosis is reported for the first time. The management of this disease is comprehensive and primarily centered on surgical intervention. Adjuvant treatment remains controversial. Patients typically have a good prognosis even with metastasis. Furthermore, due to the rarity of this disease, there is a lack of established standardized diagnostic and therapeutic criteria. The differential diagnosis of retroperitoneal tumors in women still requires consideration of ESS. In conclusion, further research involving a larger number of cases is necessary to establish consistent diagnostic approaches and formulate treatment guidelines.

## Acknowledgments

This work was supported by the Province Key Research and Development Project of Shandong (2016WS0562), CSCO-HengRui Tumor Research Fund (Y-HR2018-200), the Medical Science and Technology Project of Shandong (2016WSA18028, 2011HZ097), the Natural Science Foundation of Shandong (ZR2012HM010), the Province Key Research and Development Project of Shandong (2018GSF118238).

## Author contributions

**Software:** Yaoyao Fang, Yingchun Zheng, Sijia Bo, Lei Sui, Ting Zhu.

**Validation:** Yaoyao Fang, Xiaoli Zhou, Jing Bai, Qiuju Lin, Yingchun Zheng, Sijia Bo, Lei Sui, Ting Zhu, Nuo Bai, Li Sun.

**Data curation:** Xiaoli Zhou, Jing Bai, Qiuju Lin.

**Formal analysis:** Xiaoli Zhou, Qiuju Lin, Li Sun.

**Investigation:** Xiaoli Zhou, Jing Bai, Qiuju Lin.

**Conceptualization:** Jing Bai, Li Sun.

**Writing—original draft:** Jing Bai.

**Writing—review & editing:** Jing Bai, Li Sun.

**Supervision:** Nuo Bai.
